# The NADPH oxidase 4 is a major source of hydrogen peroxide in human granulosa-lutein and granulosa tumor cells

**DOI:** 10.1038/s41598-019-40329-8

**Published:** 2019-03-05

**Authors:** Theresa Buck, Carsten Theo Hack, Dieter Berg, Ulrike Berg, Lars Kunz, Artur Mayerhofer

**Affiliations:** 1 Biomedical Center Munich (BMC), Cell Biology, Anatomy III, Ludwig-Maximilians-Universität München, 82152 Planegg, Martinsried Germany; 2 A.R.T. Bogenhausen, 81675 Munich, Germany; 3 Division of Neurobiology, Department Biology II, Ludwig-Maximilians-Universität München, 82152 Planegg, Martinsried Germany

## Abstract

H_2_O_2_ is a reactive oxygen species (ROS), which can diffuse away from its site of generation and may act as a cell-to-cell signaling factor. The mechanisms responsible for the generation of H_2_O_2_ in human ovarian follicles and possible signaling role(s) of H_2_O_2_ are not well known. We identified a source of H_2_O_2_, the enzyme NADPH oxidase (NOX) 4, in isolated differentiated, *in-vitro* fertilisation-derived human granulosa-lutein cells (GCs), in proliferating human granulosa tumour cells (KGN), as well as *in situ* in cells of growing ovarian follicles. H_2_O_2_ was readily detected in the supernatant of cultured GCs and KGN cells. H_2_O_2_ levels were significantly lowered by the NOX4 blocker GKT137831, indicating a pronounced contribution of NOX4 to overall H_2_O_2_ generation by these cells. We provide evidence that extracellular H_2_O_2_ is taken up by GCs, which is facilitated by aquaporins (peroxiporins). We thus conclude that GC-derived H_2_O_2_ might act as autocrine/paracrine factor. Addition of H_2_O_2_ increased MAPK-phosphorylation in GCs. Moreover, reducing H_2_O_2_ production with GKT137831 slowed proliferation of KGN cells. Our results pinpoint NOX4 and H_2_O_2_ as physiological players in the regulation of GC functions.

## Introduction

Reactive oxygen species (ROS), including hydroxyl, peroxyl and other molecules, function as physiological regulators of hormone actions and signal transduction pathways, to affect processes in the female gonad^1–3^. Studies in the rat revealed a role in ovulation^4^. In the corpus luteum they are beneficial for steroid production^5^. Furthermore, it is becoming clear that specifically hydrogen peroxide (H_2_O_2_) plays fundamental roles in cell proliferation, migration and metabolism, as well as cell death^3^.

ROS are a double-edged sword and a dysregulation of their levels may also cause or accelerate pathological conditions and oxidative stress, associated, for instance, with impaired oocyte quality, follicular atresia and negative outcome of assisted reproductive techniques (ART)^6–8^.

Several intracellular sources of ROS and modes of their generation exist^1,3^. However, in the human ovary neither those sources, nor the mechanisms of their actions are well known. It was shown that ROS levels in *in-vitro* fertilization (IVF)-derived, cultured human granulosa-lutein cells (GCs) are increased upon uptake and intracellular metabolism of the catecholamines dopamine and norepinephrine^9–11^. ROS generation in GCs was also increased upon activation of the epidermal growth factor, as well as of the pigment epithelium derived growth factor^12,13^.

During these studies it became clear that yet a further ROS source exists in GCs, namely NADPH oxidases (NOX); see e.g.^1,14^. These are multicomponent, membrane-bound enzymes, of which seven members are known, NOX1-5 and dual oxidases (DUOX) 1 and 2^15,16^. Of those, *NOX4* and *5* were previously described in human GCs by RT-PCR^12^, *NOX1-3* were not found and *DUOX1* and *2* have not been studied yet.

The main product of NOX4 is H_2_O_2_, which is long-lived and the only membrane-permeable ROS^17^. Therefore, it may serve as an autocrine/paracrine signaling molecule. In accordance with this statement, it was found in human follicular fluid (FF)^18^. Uptake of extracellular H_2_O_2_ can be facilitated by aquaporins (AQPs), besides H_2_O_2_ diffusion through the plasma membrane^19^. Interestingly, a recent study did not only confirm expression of NOX4 in IVF-derived GCs, but demonstrated a decrease of NOX4 levels in GCs from women older than 40 years^20^. This correlates with the naturally occurring decline in fertility with age and might imply importance of high NOX4 levels in physiological processes in the ovarian follicle. Beneficial and physiological roles of H_2_O_2_ and NOX4 have also been reported for the vasculature^21^.

We examined NOX enzymes in differentiated GCs and in proliferating human granulosa tumor cells, KGN^22^. We focused on NOX4 as a H_2_O_2_-producing enzyme, studied involvement of AQP channels in the cellular uptake of H_2_O_2_^1,19^ and explored potential consequences of action.

## Results

### Identification of NADPH-oxidases in human GCs and ovarian sections

**Figure 1:**
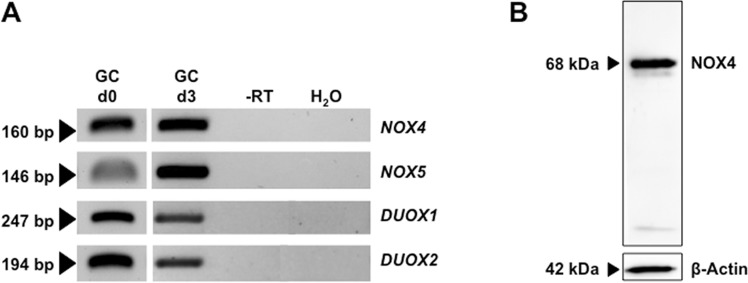
NOX expression in GCs *in vitro*. (**A**) RT-PCR identified *NOX4*/5 and *DUOX1/2* in cultured GCs after isolation (d0) and on culture day 3 (pooled GCs from 2–5 individuals). Controls including RNA (-RT) and H_2_O instead of cDNA (H_2_O) were negative. The displayed figure was cropped from original gel images, which are part of the supplementary data. (**B**) NOX4 protein was detected in cultured GCs by immunoblotting (anti-NOX4 antibody from ProSci). Controls without primary antibody were negative.

**Figure 2:**
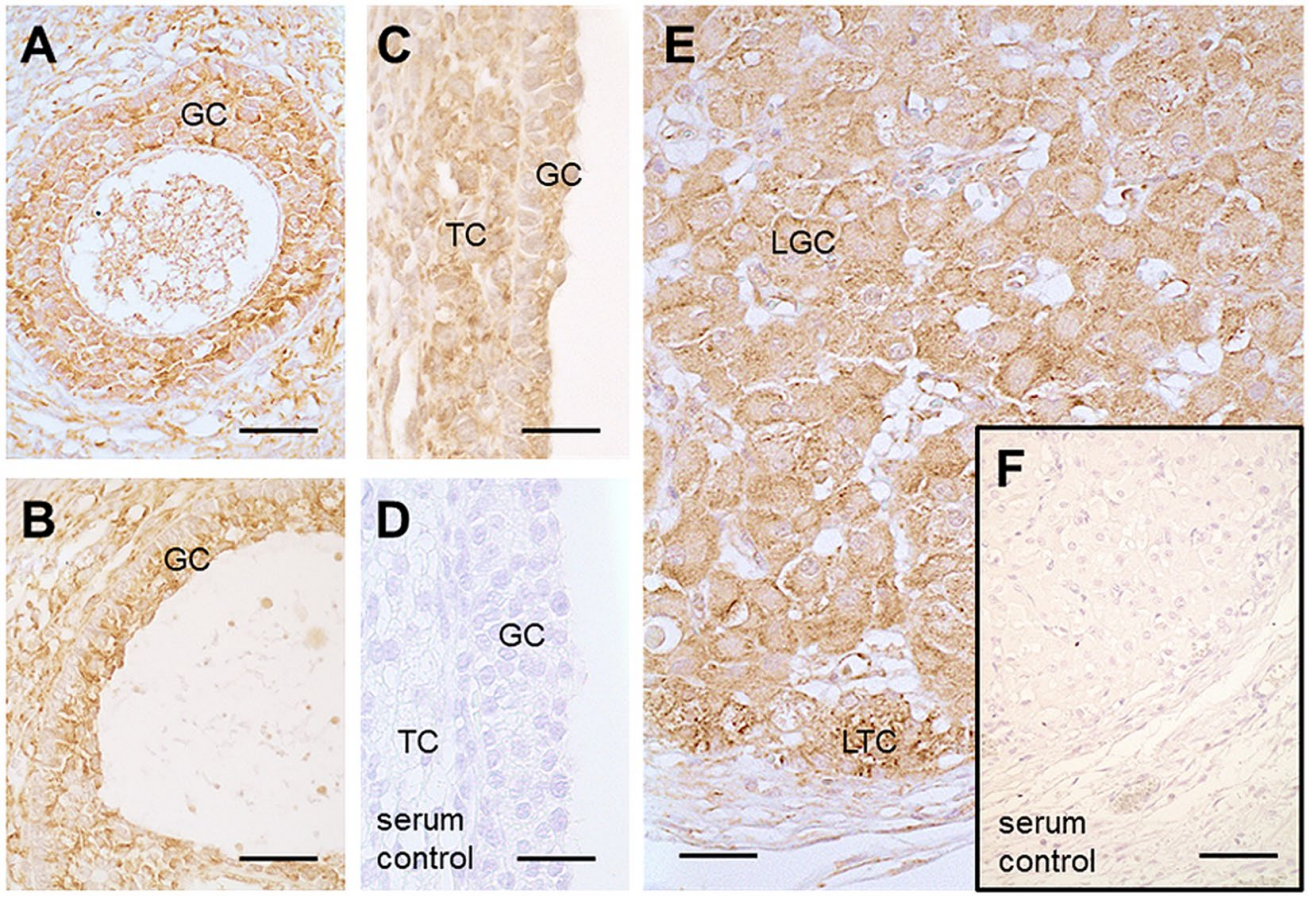
Presence of NOX4 in human ovarian tissue. Immunohistochemistry using human ovarian sections and an anti-NOX4 antibody from ProSci showed positive staining for NOX4 in granulosa (GC) and theca cells (TC) of a secondary follicle (**A**), of a small antral follicle (**B**), of a large antral follicle (**C**) as well as in luteinized GCs (LGC) and luteinized TCs (LTC) of the corpus luteum (**E**). Serum controls lacked first antibody (**D** and **F**). Scale bars: A–E = 30 µm, F = 50 µm.

RT-PCR followed by sequencing revealed that GCs on the day of isolation (day 0 = d0) and on culture day 3 (d3) express *NOX4*, *NOX*5, *DUOX1* and *DUOX2* (Fig. 1A). This experiment was repeated using n = 5 independent pooled GCs preparations. NOX4 protein was detected in three GC preparations by Western blotting (Fig. 1B; NOX4: 68 kDa). Immunohistochemistry revealed NOX4 in preantral and antral follicles (Fig. 2A–C) and the corpus luteum of the human ovary (Fig. 2E). Follicular granulosa cells, theca cells and small and large luteal cells were stained (Fig. 2). This staining pattern was also obtained with another antibody (Supplementary Fig. S5). Specificity was shown by pre-adsorption of one of the antibodies with a blocking peptide (Supplementary Fig. S5), or replacing the antibody by serum (Fig. 2D,F).

### H_2_O_2_ production by NOX4

**Figure 3:**
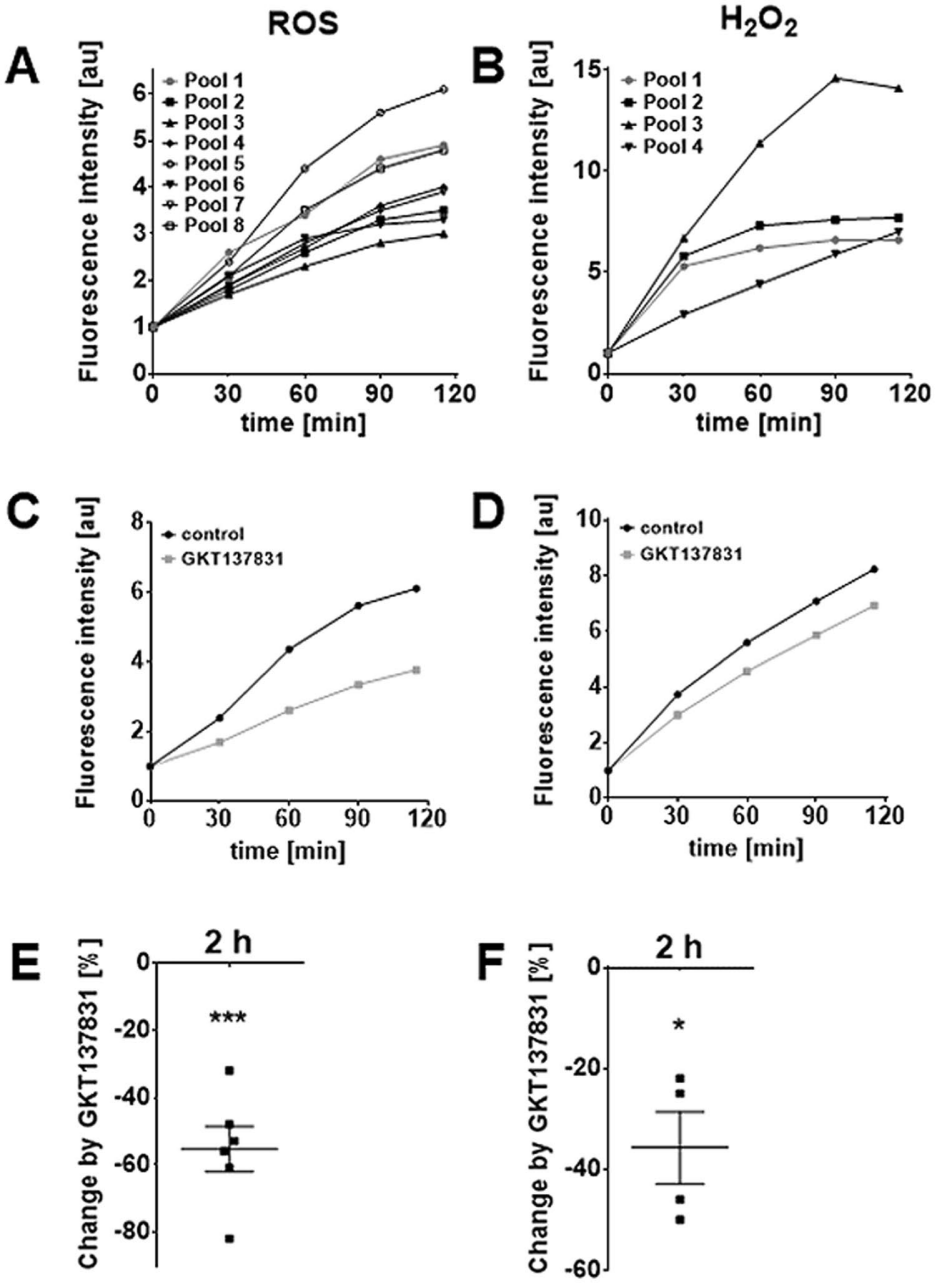
GCs are a source of H_2_O_2_ and other ROS produced by NOX4. Basal production of ROS (identified by the indicator dye H_2_DCFDA, n = 8) and specifically of H_2_O_2_ (detected by Amplex^®^ Red, n = 4) was measured on culture day 2 to 4 for 2 h (**A**,**B**). For clarity, results are presented as mean only (six technical repetitions) and as fold-change relative to the intensity at t = 0. The NOX4 blockerGKT137831 (20 µM) reduced the production of ROS/H_2_O_2_. One representative measurement for both is shown in (**C**,**D**). Blocking NOX4 significantly reduced production of ROS (**E**; by 55%; n = 6) and H_2_O_2_ (**F**; by 36%; n = 4) after 2 h (mean ± SEM). One-sample *t*-tests (two-tailed; *p < 0.05; ***p < 0.001).

Fluorometrical measurements of ROS employing H_2_DCFDA (n = 8 GC pools) and detection of H_2_O_2_ by Amplex^®^ Red reagent (n = 4) confirmed basal generation of H_2_O_2_ and other ROS (Fig. 3). Superoxide was detected only in small amounts (Supplementary Fig. S1; n = 4). An increase in ROS, and specifically in H_2_O_2_ was seen in all independent measurements in the course of 2 h (Fig. 3A,B). The NOX4-blocker GKT137831 significantly reduced ROS generation after 2 h by 55% and H_2_O_2_ production by 36%, respectively (Fig. 3C–F). The blocker did not affect cell viability (24 h; confirmed by determination of ATP content, n = 3) and morphology (Supplementary Fig. S2A,B).

### Influence of FSH and hCG on NOX enzyme levels

**Figure 4:**
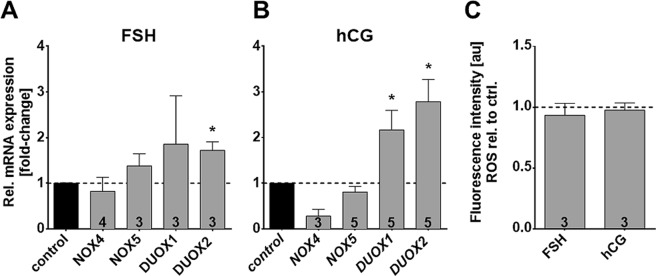
Hormonal stimulation significantly increases levels of *DUOX*, but does not elevate basal ROS production in GCs. Results of qRT-PCR experiments showed that mRNA expression levels of *NOX5/DUOX1/2* were elevated after hormonal stimulation, but not all reached statistical significance (**A**,**B**). Results (mean ± SEM) shown were normalized to the geometric mean of the reference genes *RPL19, UBC, PPIA* and *GAPDH*. (**C**) Measurements of ROS production using H_2_DCFDA did not show any difference after 2 h measurement when treated with the hormones. Numbers of repeated experiments are shown inside the columns. Mean ± SEM; one-sample *t*-tests (two-tailed; *p < 0.05)

Luteal cells and cultured GCs express receptors for FSH and LH/hCG^23^. Addition of either hormone for 24 h did not affect levels of *NOX4* and *NOX5*, but significantly increased *DUOX1* and *DUOX2* (Fig. 4A,B). FSH induced a 1.7-fold increase in *DUOX2* expression. *DUOX1* was increased 2.2-fold and *DUOX2* 2.8-fold by hCG. Overall ROS generation after 24 h of stimulation with FSH/hCG was not increased (Fig. 4C).

### Uptake of H_2_O_2_ by GCs

**Figure 5:**
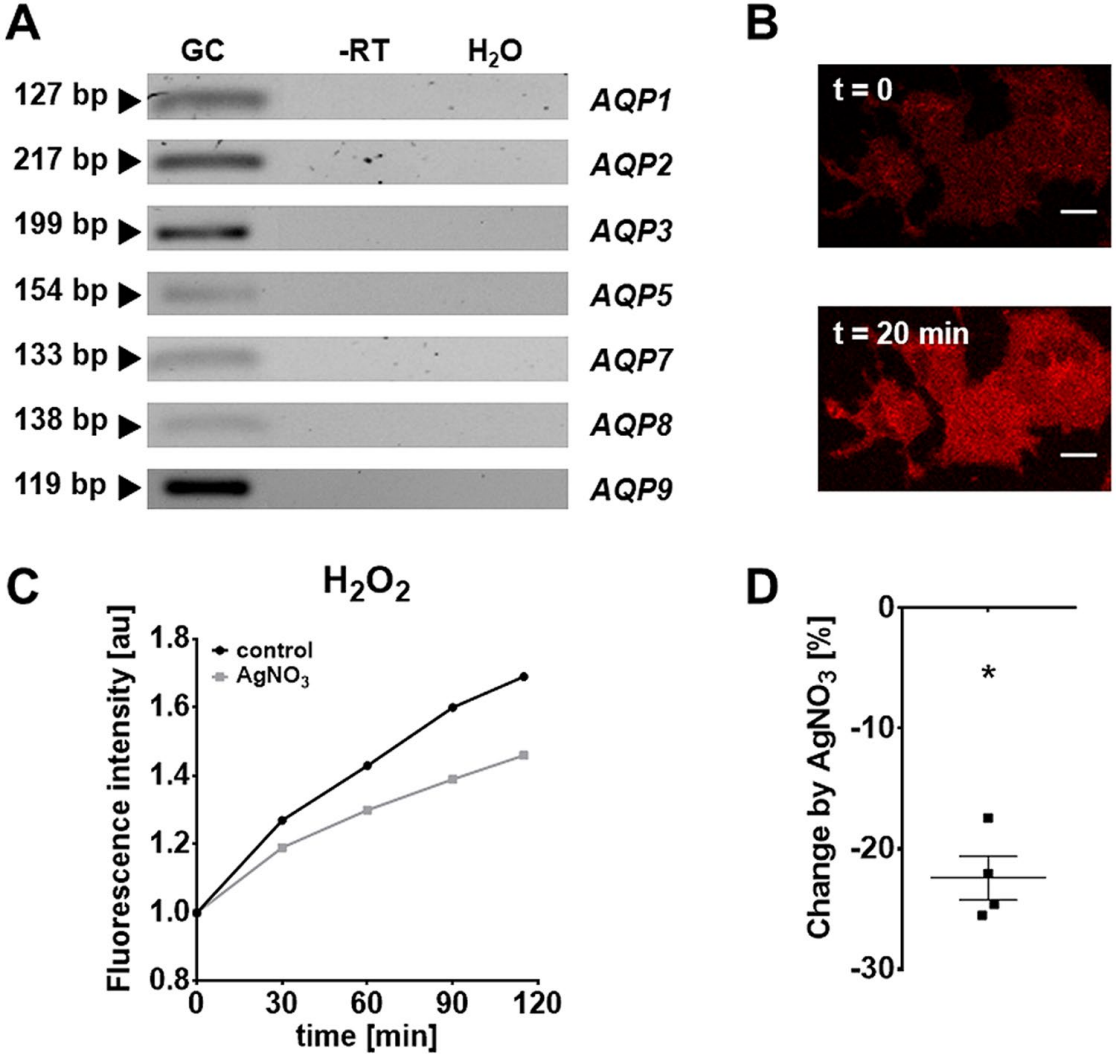
H_2_O_2_-transporting aquaporins in GCs. (**A**) RT-PCR identified *AQP1, 2, 3, 5, 7, 8* and *9*. All controls, including RNA (-RT) and H_2_O instead  cDNA (H_2_O), were negative. The displayed figure was cropped and the original gel images are part of the supplementary data. (**B**) Fluorescence image of GCs preloaded with PO1 at the start of the monitoring (t = 0) and 20 min after addition of H_2_O_2_ (final concentration: 100 µM). Scale bar = 10 µm. (**C**,**D**) The aquaporine blocker AgNO_3_ (500 nM) significantly decreased H_2_O_2_ production by 22 % (after 2 h). Data are shown as mean of six technical repetitions of a single measurement (relative to the intensity at t = 0; n = 1). (**D**) Percentage decrease of four measurements compared to control. One-sample *t-*test (two-tailed; *p < 0.05).

Several aquaporins (AQP3, 8 and 9) are implicated in the transport of H_2_O_2_ and therefore called peroxiporines. The expression of some AQPs in GCs was reported^24–30^. We confirmed expression of *AQP1-3*, *5*, and *7–9* by RT-PCR (Fig. 5A). *AQP4* and 6 were not found. PO1, a cell permeable fluorescent probe visualizes intracellular H_2_O_2_, thereby allows to monitor its cellular uptake. Both, confocal microscopy (n = 3) and quantitative fluorometric evaluations (n = 4) showed that extracellular H_2_O_2_ was rapidly taken up by GCs. The increase of intracellular H_2_O_2_ was significantly reduced in the presence of AgNO_3_, which can block AQPs^31^ (Fig. 5B–D). The blocker used at 500 nM did not affect cell viability (2 h; confirmed by determination of ATP content, n = 3; Supplementary Fig. S3).

### Roles of H_2_O_2_ in GCs and KGN

To explore specific roles of NOX4-derived H_2_O_2_, two strategies were employed. Consequences of the NOX4 blocker GKT137831, thereby lowering production of H_2_O_2_, and consequences of direct addition of H_2_O_2_ were studied.

**Figure 6:**
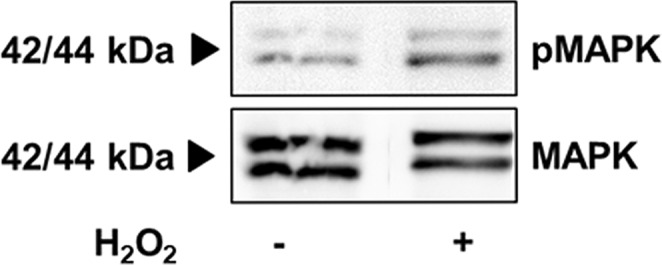
H_2_O_2_ increases phosphorylation of MAPK. (**A**) WB membrane shows the expression of MAPK and pMAPK of treated and untreated GCs on culture day 1 of a representative example (arrows show expected size at 42 and 44 kDa; two repetitions). The displayed figure was cropped and the original gel images are part of the supplementary data.

H_2_O_2_ treatment (2 h) resulted in an increased phosphorylation of MAPK (representative example shown in Fig. 6, n = 2).

**Figure 7:**
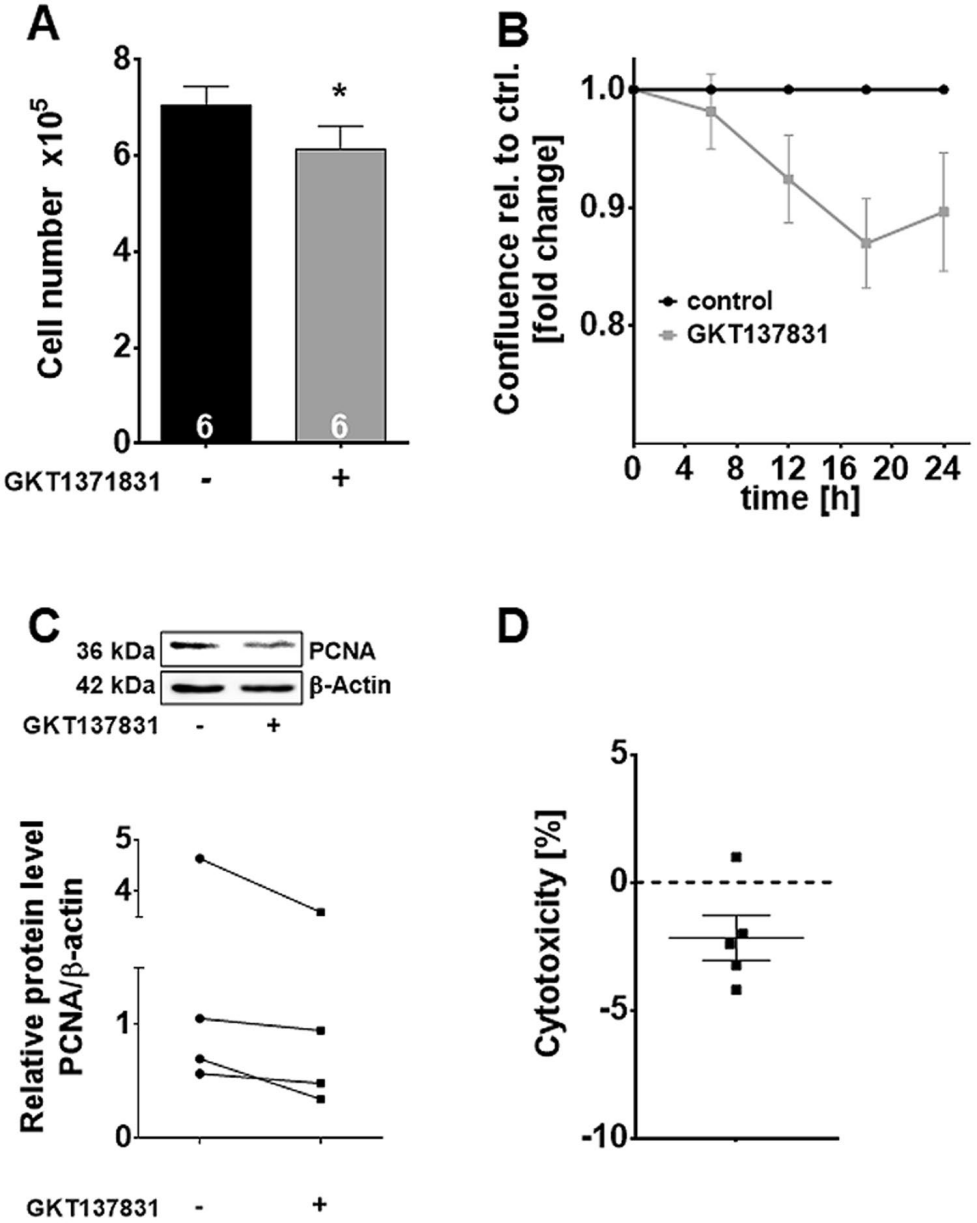
Actions of a NOX4 blocker in KGN. KGN treatment with GKT137831 resulted in a significant reduction in cell number (**A**; n = 6) and confluence (**B**; n = 4), as well as reduced PCNA content. The figure was cropped and the original gel images are shown in the supplementary data (**C**) PCNA in KGN compared to control (blotted membrane of one representative example is shown). Diagram shows PCNA levels of control versus treated cells normalized to β-actin (n = 4). (**D**) Treatment with GKT137831 had no cytotoxic effect in KGN compared to control (LDH cytotoxicity assay; n = 5). One-sample *t*-tests (two-tailed; *p < 0.05).

Because primary GCs do not proliferate *in vitro*, we studied proliferation in a human granulosa tumor cell line (KGN). In KGN, we confirmed expression of *NOX4* by RT-PCR and a significant reduction of H_2_O_2_ production by NOX4 blocker (Supplementary Fig. S4A,B). Treatment with GKT137831 did not affect KGN viability (Fig. 7D; LDH assay, n = 5), but significantly reduced cell number (Fig. 7A; n = 6) and confluence over 24 h (Fig. 7B; n = 4). Accordingly, expression of the proliferation marker PCNA was reduced, as shown by Western blotting (Fig. 7C; n = 4).

## Discussion

The results of this study show that NOX4 is a major producer of H_2_O_2_ in human GCs *in vitro*. *NOX4* levels are not under the control of FSH or LH/hCG in GCs. H_2_O_2_ can potentially act as a cell-cell signaling factor and AQPs may be involved in facilitating membrane transport of this ROS. The consequences of H_2_O_2_ signaling remain to be fully explored, yet expression of NOX4 *in situ*, and in growing follicles, as well as our results in KGN indicate a role in proliferation of ovarian cells.

There are few studies examining NOX enzymes, their specific ROS products and their roles in the ovary^16,32–34^. *NOX1-3* were not found in GCs, but previous studies showed expression of *NOX4* in human GCs^12,20,34^. This was confirmed by our study. NOX4 protein was further detected by immunohistochemistry in granulosa, theca and luteal cells of the human ovary. These results are in line with previous reports^35–37^.

Besides *NOX4*, we also detected *NOX5*, *DUOX1* and *2. NOX5* was described in a previous study^12^. This human specific enzyme is absent in mice^38^, and generates superoxide, which is rapidly dismutated to H_2_O_2_. DUOX1/2, as well as NOX4 generate H_2_O_2_^32^. This might partially explain why we only found traces of superoxide, but abundant H_2_O_2_. H_2_O_2_ could be the result of NOX4, and DUOX1 and DUOX2 activities. Experimental studies on NOX5, as well as DUOX1/2 are limited by the lack of specific antibodies and pharmacological tools^32^, yet such tools exist for NOX4.

In the vascular system NOX4 activity accounts for about one-third of endothelial H_2_O_2_ formation^17^ and a study in mice reported a protective role of NOX4 for vascular function^21^. We applied the Amplex^®^ Red Hydrogen Peroxide/Peroxidase Assay Kit, a purely extracellular H_2_O_2_ detection method, and a cell permeable boronate compound (PO1) specifically activated by intracellular H_2_O_2_. The measurements confirmed the presence of intra- and extracellular H_2_O_2_. The results indicate that GCs, as well as KGN, can produce H_2_O_2_, which can leave its cellular sites of generation. In combination with experiments using a NOX4 blocker they revealed a 36% contribution of NOX4 to overall H_2_O_2_ generation. Results are in line with a report on the presence of H_2_O_2_ in FF^39^.

As an extracellularly released factor, H_2_O_2_ may act on neighboring cells or oocytes. We examined, whether, as a prerequisite for such autocrine/paracrine H_2_O_2_ actions, it may be able to enter GCs. This is assumed to be a criterion for interaction with intracellular targets. Our studies using PO1 showed that this is the case. AQP3, *8* and 9 reportedly facilitate transport of H_2_O_2_ and are referred to as peroxiporins^29,30^. In line with previous studies, we identified *AQP3*, *8* and 9 in GCs^24^. Blocking AQPs with AgNO_3_ in the presence of extracellular H_2_O_2_ supports a significant contribution of these channels in the uptake of H_2_O_2_. Whether they are also involved in the release of this ROS, remains to be studied.

How NOX4 expression is regulated also remains to be shown. The human ovarian samples available for immunohistochemistry showed expression in growing follicles, yet NOX4 expression in resting follicles could not be evaluated. The studies in cultured GCs, derived from preovulatory follicles, are in line with the notion that NOX4 is a constitutive enzyme, as our results did not reveal that FSH or hCG affect its level. FSH-, and LH-receptors were present and functional, as concluded from the increase in *DUOX1/2* by this treatment. The increase was, however, without measurable consequences with regard to overall ROS or specifically H_2_O_2_ production.

The widespread expression of NOX4 in the normal human ovary may imply important physiological roles. As previously described in the rat, H_2_O_2_ caused phosphorylation of 42/44 MAPK^4^. This is also in line with a recently published paper demonstrating the necessity of NOX-derived ROS for the EGF/MAPK signaling pathway in uterine leiomyomas^40^. We found increased phosphorylation of 42/44 MAPK after direct addition of H_2_O_2_ to GCs. Additional studies are required to further examine downstream pathways.

As proliferation is crucial for follicular growth and NOX4 was detected in growing follicles, we studied a possible involvement of NOX4 in GC proliferation by using KGN cells as a model system. These cells, derived from a human GC tumor^22^, proliferate in contrast to primary GCs. Blocking NOX4 reduced H_2_O_2_ production, cell number and confluence. As no signs of cytotoxicity were detected and expression of the proliferation marker PCNA was reduced, our results indicate that NOX4-derived H_2_O_2_ is involved in cell proliferation. The precise mechanisms of action and possible other roles remain to be studied.

## Materials and Methods

### Human GC isolation, culture and treatment, KGN culture

Follicular fluid (FF) containing GCs was derived from more than 300 patients (age range 28 to 40 years) undergoing IVF due to poor quality of sperm or restricted tubal patency. The stimulation protocols (i.e. the “long” protocol), were described^9,10,41–44^. The ethics committee of the Ludwig-Maximilians University Munich approved the use of follicular aspirates and GCs for scientific experiments (Project 323-05). A written informed consent of the patients was obtained, samples and clinical information were anonymized. All experiments were performed in accordance with relevant guidelines and regulations (including all laboratory and biosafety regulations). The FF from two to five patients were pooled for each preparation of GCs, following a method^45^, which utilizes a cell strainer (40 μm; BD, Franklin Lakes, NJ, USA) for filtration, as described previously^9,10,46^. Cells were cultured in DMEM/Ham’s F12 medium supplemented with penicillin (100 U/ml), streptomycin (100 μg/ml) and 10% fetal calf serum (FCS; all from PAA) for up to 4 days at 37 °C and with 5% CO_2_. For all experimental treatments, DMEM/Ham’s F12 medium without FCS was used.

The KGN cell line was obtained from RIKEN BioResource Center, and cultured as described^22^ in DMEM/Ham’s F12 medium supplemented with penicillin (100 U/ml), streptomycin (100 μg/ml) and 10% FCS.

For the experiments 20 µM GKT137831 (Cayman Chemical, Michigan, USA), 10 mM 3-Amino-1,2,4-triazole (3-AT) (Sigma-Aldrich, St. Louis, Missouri, USA), 1 IU/ml human recombinant FSH (Cedarlane, Burlington, NC, USA) or 10 IU/ml hCG (Sigma-Aldrich) were used, together with the appropriate corresponding solvent controls (including DMSO, PBS and 0.01 M NaH_2_PO_4_; all from Sigma-Aldrich), diluted in DMEM/Ham’s F12 medium (Thermo Fisher Scientific, Darmstadt, Germany).

### Reverse transcription-PCR and quantitative RT-PCR (qRT-PCR)

Total RNA was isolated using the RNeasy Plus Micro Kit (Qiagen, Hilden, Germany). RNA concentration and purity was determined using a NanoDrop® spectrophotometer (Thermo Fisher Scientific). cDNA was produced using random pentadecamer primers (metabion international AG; Munich, Germany) and Superscript II Reverse Transcriptase (Life Technologies). RT-PCR was arranged with different oligomer primers (metabion international AG) listed in Table 1 and GoTaq DNA Polymerase Kit from Promega (Mannheim, Germany), amplicons were visualized by agarose gel electrophoresis and were verified by sequencing.

**Table 1:** List of oligonucleotide primers used for PCR studies.

Gene	Accession number	Primer sequence (5′-3′)	Product size [bp]
*NOX4*	NM_001143837.1	Forward: ccg aac act ctt ggc tta cc Reverse: gtt gag ggc att cac cag at	160
*NOX5*	NM_024505.3	Forward: gct gtc gag gag tgt gac aa Reverse: gct cag agg caa aga tcc tg	146
*DUOX1*	NM_175940.2	Forward: cct ctg agc agt tcc tgt cc Reverse: aaa tcc cgc aca tct tca ac	247
*DUOX2*	NM_014080.4	Forward: ggc aaa ttc tcc cgt aca ga Reverse: agc tgg gat agg tcc tgg tt	194
*AQP1*	NM_001329872.1	Forward: atc ttc cgt gcc ctc atg ta Reverse: cac cat cag cca ggt cat tg	127
*AQP2*	NM_000486.5	Forward: ctg gct gtc aat gct ctc ag Reverse: agc ggg cag gat tca tag a	217
*AQP3*	NM_001318144.1	Forward: ttt ggc ttt gct gtc act ct Reverse: gcc aga ttg cat cat aat aca gc	199
*AQP4*	NM_004028.4	Forward: gat cag cat cgc caa gtc tg Reverse: gcg gta aga ttt cca tga acc at	141
*AQP5*	NM_001651.3	Forward: gtc aac gcg ctc aac aac aa Reverse: tga cag aca ggc caa tgg ac	154
*AQP6*	NM_001652.3	Forward: cgt agg ctc cca cat ctc tc Reverse: aga cac tgt tcc gga cca c	149
*AQP7*	NM_001318156.1	Forward: tga cct ttg cta act gtg cg Reverse: aag tgg aga atg gcc gtg ta	133
*AQP8*	NM_001169.2	Forward: gtc tgg agg ctg cat gaa tc Reverse: cca atg aag cac cta atg agc a	138
*AQP9*	NM_020980.4	Forward: cgg tgt ctc tgg tgg tca Reverse: cca caa agg ctc cca aga ac	119
*RPL19*	NM_000981.3	Forward: agg cac atg ggc ata ggt aa Reverse: cca tga gaa tcc gct tgt tt	199
*UBC*	AB_362574.1	Forward: gcc tta gaa ccc cag tat cagReverse: aag aaa acc agt gcc cta gag	74
*PPIA*	KJ_905864.1	Forward: aga caa ggt ccc aaa gacReverse: acc acc ctg aca cat aaa	118
*GAPDH*	NM_002046.6	Forward: gtc ttc act acc atg gag aag gReverse: tca tgg atg acc ttg gcc ag	197

For qRT-PCR studies duplicates were performed and the QuantiFast SYBR Green PCR Kit (Qiagen) was applied using following protocol in a LightCycler^®^ 96 System (Roche Diagnostics, Penzberg, Germany): heat activation (95 °C, 5 min), 40 cycles denaturation (95 °C, 10 s) and annealing/extension (60 °C, 30 s) followed by melting (95 °C, 10 s; 65 °C, 60 s and 97 °C, 1 s) and cooling-down (37 °C, 30 s). Negative controls included RNA, added instead of cDNA (-RT), and H_2_O, instead of cDNA. Quantitative results were calculated according to the 2^−ΔΔCq^ method^47^ and normalized for the geometric mean of the reference genes *RPL19, UBC, PPIA* and *GAPDH*, as well as to the respective, untreated control. Results are depicted as means ± SEM.

### Western blot

The method was performed as described^46^. Protein samples were analyzed (7,5 µg of GCs on culture day 2 and 10 µg of KGN) with anti-NOX4 rabbit polyclonal antibody (Cat#: 7927, ProSci; Fort Collins, CO, USA), anti-PCNA (Cat#: 610664; BD), anti-MAPK (Cat#: 4695, Cell Signaling), anti-pMAPK (Cat#: 9106, Cell Signaling), as well as anti-β-Actin mouse monoclonal antibody (Cat#: A5441, Sigma-Aldrich). Secondary antibodies were conjugated with horseradish peroxidase (Jackson, Bar Harbor, Maine, USA).

### Immunohistochemistry

Immunohistochemistry was performed using the same archival human ovarian sections as described^46^. A goat polyclonal IgG raised against human NOX4 (NB110-58849, Novus Biologicals, Littleton; CO; USA), and the same antibody used also in Western blot studies, as well as biotinylated secondary antibodies (Jackson, Bar Harbor, Maine, USA) were employed. The primary antiserum was pre-adsorbed by a corresponding blocking peptide (NB110-58849PEP, Novus Biologicals). As a further control the antiserum was replaced by non- immune serum. Hematoxylin-eosin-staining was performed and images were taken with an Axiovert microscope (Zeiss, Jen, Germany).

### Measurement of ROS generation

ROS generation was measured with the 2-,7-dichlorodihydrofluorescein diacetate (H_2_DCFDA) method, as described^9–11^. The cell permeant reagent H_2_DCFDA is a fluorogenic dye, which measures hydroxyl, peroxyl and other ROS within cells. After diffusion into the cell, it is deacetylated by cellular esterases to a non-fluorescent compound, which is then oxidized by ROS into 2′, 7′ –dichlorofluorescein (DCF). DCF is a highly fluorescent compound, which can be detected by fluorescence spectroscopy. GCs of independent preparations of cells from two to five patients were seeded into black 96-well plates (Nunclon Delta Surface; six technical repetitions for each group), cultured for 24 h, preloaded with H_2_DCFDA (10 µM) for 30 min and fluorescence levels were measured at 485 nm excitation/ 520 nm emission in a fluorometer (FLUOstar Omega; BMG labtech, Ortenberg, Germany) for 2 h at 37 °C. The final values were statistically analyzed and depicted relative to the value at t = 0.

### Measurement of H_2_O_2_ generation

The generation of H_2_O_2_ was measured using an Amplex^®^ Red Kit (Invitrogen), as described^11^. Briefly, Amplex® Red reagent (10-acetyl-3,7-dihydroxyphenoxazine) is a colorless substrate, which reacts with H_2_O_2_ with a 1:1 stoichiometry to produce fluorescent resorufin. The method is highly sensitive and as little as 10 picomoles of H_2_O_2_ in a 100 µL volume (100 nM) can be detected. GCs were seeded as described for ROS measurement and 1.5 × 10^4^ KGNs/ well, respectively. Amplex^®^ Red reagent was used in a final concentration of 2.5 µM and fluorescence levels were measured at 544 nm excitation/590 nm emission in a fluorometer (BMG labtech) for 2 h at 37 °C. The mean values were normalized to the starting point value.

### Measurement of H_2_O_2_ uptake in GCs

Intracellular H_2_O_2_ levels in cultures of human GCs were quantified in black 96-well microplates (Nunclon Delta Surface) using the cell permeable boronate-based fluorescent H_2_O_2_-probe Peroxy Orange 1 (PO1) (Tocris, Bristol, UK) by exogenous preloading as described^48^. Briefly, GCs were seeded one day prior to measurements. Culture medium was replaced with extracellular (EC) fluid buffer (140 mM NaCl, 3 mM KCl, 1 mM MgCl_2_, 1 mM CaCl_2_, 10 mM HEPES and 10 mM glucose; pH 7.4) and cells were preloaded for 20 min with 1 µM PO1 and/or 500 nM AgNO_3_ (Honeywell, Seelze, Germany). The concentration (500 nM) was chosen in pilot experiments, because it was not toxic to GCs (see Supplementary Figure S3) and effectively blocked the measurable increase in PO1- and DCF-fluorescence. Fluorescence was determined using a microplate reader (BMG labtech) after replacing the staining solution with 100 µM H_2_O_2_ in EC buffer (544 nm excitation/590 nm emission).

In addition, fluorescent PO1 was examined using a Leica TCS SP8 confocal microscope (Leica, Wetzlar, Germany). GCs were cultured on a µ-Dish ^35mm, high^, ibiTreat (ibidi GmbH, Martinsried, Germany) for 2 days. Prior to microscopy, culture medium was replaced by 1 µM PO1 diluted in EC buffer and after 20 min H_2_O_2_ (end concentration: 333 µM) was added. Fluorescence imaging was performed at t = 0 and t = 20 min at 37 °C (552 nm excitation/562–650 nm emission via HyD detector).

### Cell viability assay

Cellular ATP content^49^ correlates with viability and was determined using CellTiter-Glo Luminescent Cell Viability Assay (Promega; Mannheim, Germany), as described^49^. The assay system is based on the properties of a thermostable luciferase to enable reaction conditions, which generate a stable luminescent signal while inhibiting endogenous enzymes released during cell lysis (e.g., ATPases). The luminescence was measured in a luminometer (BMG labtech).

### Lactate dehydrogenase (LDH) assay

LDH indicates damaged cell membranes and thus cytotoxicity of GKT137831 was measured in KGN (1.5 × 10^4^ cells/well) using a LDH cytotoxicity assay kit (Thermo Fisher Scientific), as described^46^. LDH is a cytosolic enzyme released upon plasma membrane damage into the cell culture media. Extracellular LDH can be quantified by a coupled enzymatic reaction, in which LDH catalyzes the conversion of lactate to pyruvate via NAD^+^ reduction to NADH. Diaphorase then uses NADH to reduce a tetrazolium salt to a red formazan product, which can be measured at 490 nm. The level of formazan formation is directly proportional to the amount of LDH released into the medium, which is indicative of cytotoxicity.

### Confluence and cell counting

The methods were described previously^46^. Briefly, 1.5 × 10^5^ KGNs/dish were monitored for 24 h by taking time-lapse pictures every 20 min under culture conditions. Confluence values were determined by open source software ImageJ (Fiji) and depicted as fold change to control and delta t_0_ subtracted. Cells were counted before and after treatment (t = 0 and 24 h) by automated cell counter (Biozym).

### Statistics

GraphPad Prism 6.0 Software (GraphPad Software Inc., San Diego, CA, USA) was used to perform one-sample *t*-tests (two-tailed) for comparisons of −ΔΔC_q_ values and for ROS and H_2_O_2_ measurements as well as for ATP-, LDH-assay and cell number analysis. If not stated otherwise, all results are presented as means ± SEM. Corresponding *p*-values that report statistical significance (≤0.05) are individually specified in the captions.

## Supplementary information

Supplementary Information

## Data Availability

Data generated and analyzed during this study are included in this published article and its Supplementary Information files.
