# Supplementary material for: The NADPH oxidase 4 is a major source of hydrogen peroxide in human granulosa-lutein and granulosa tumor cells
Source: Sci Rep. 2019 Mar 5;9:3585. doi: 10.1038/s41598-019-40329-8 (PMC6400953; doi:10.1038/s41598-019-40329-8)
Supplement: Supplementary file 1 — Supplementary Information [file 41598_2019_40329_MOESM1_ESM.docx]

**Supplementary Information**

**The NADPH oxidase 4 is a major source of hydrogen peroxide in human granulosa-lutein and granulosa tumor cells**

**Theresa Buck^1^, Carsten Theo Hack^1^, Dieter Berg^2^, Ulrike Berg^2^, Lars Kunz^3^, Artur Mayerhofer^1*^**

^1^Biomedical Center Munich (BMC), Cell Biology, Anatomy III, Ludwig-Maximilians-Universität München, 82152 Planegg-Martinsried, Germany

^2^A.R.T. Bogenhausen, 81675 Munich, Germany

^3^Division of Neurobiology, Department Biology II, Ludwig-Maximilians-Universität München, 82152 Planegg-Martinsried, Germany

^*^Corresponding author: Artur Mayerhofer, Biomedical Center Munich (BMC), Cell Biology, Anatomy III, Ludwig-Maximilians-Universität München, Grosshaderner Strasse 9, 82152 Planegg-Martinsried, Germany

email: Mayerhofer@lrz.uni-muenchen.de

Tel.: 0049 89 2180 75859

**Supplementary Material and Methods**

**Superoxide anion detection.** Superoxide anion levels in GCs were quantified in white 96-well microplates (Nunclon Delta Surface) using a Superoxide Anion Detection Kit (Calbiochem, San Diego, CA, USA) according to the vendor’s protocol. The detection is based on the oxidation of luminol by superoxide anions resulting in the formation of chemiluminescence light. The method also utilizes a specific, non-toxic enhancer that amplifies the chemiluminescent signal. Chemiluminescence was determined using a plate-reading luminometer (BMG labtech).

**Statistics.** GraphPad Prism 6.0 Software (GraphPad Software Inc., San Diego, CA, USA) was used to perform *t*-tests (two-tailed). Corresponding *p*-values that report statistical significance (≤ 0.05) are individually specified in the captions.

**Supplementary Figures**

**
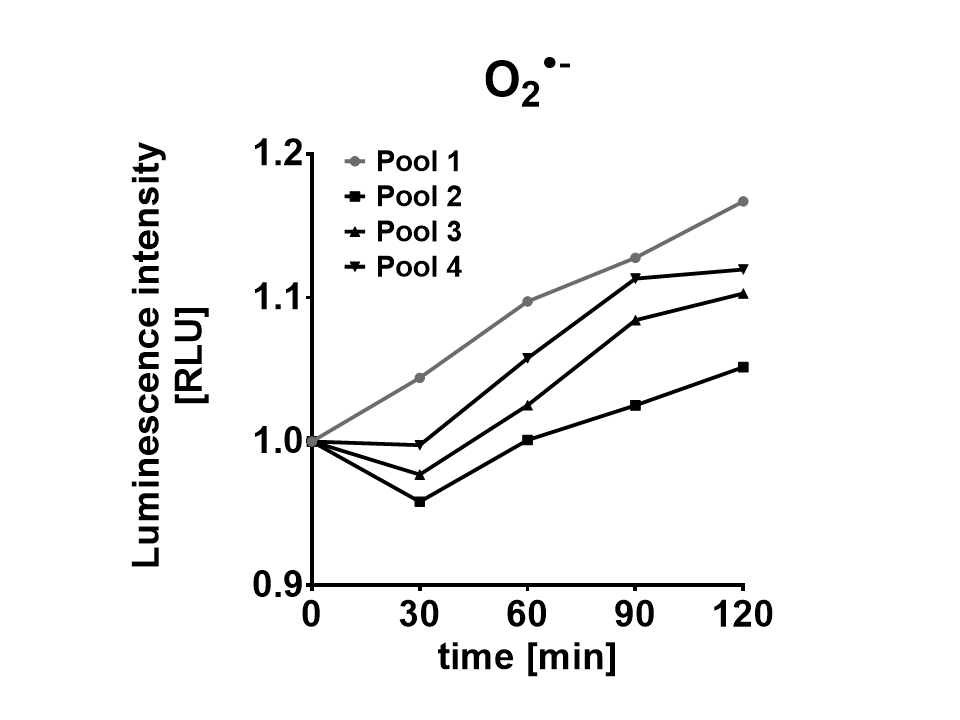
**

**Supplementary Figure S1. Superoxide anion production of GCs.** Graph shows basal production of superoxide anions determined by chemiluminescence measurements over 2 h. Each line presents an independent measurement of a patient pool. Results of the assay were not affected when either catalase (in two concentrations), 3-amino-1,2,4-triazole (3-*AT*, a blocker of catalase), or H_2_O_2_ were added to the superoxide assay.


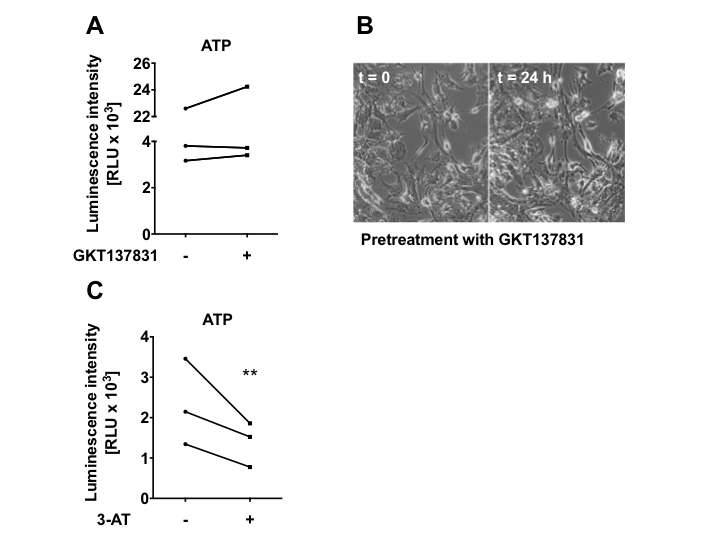


**Supplementary Figure S2. Effect of GKT137831 and 3-AT on cell viability.** 24 h pretreatment with 20 µM GKT137831 did neither alter ATP content in GCs, compared to control group (**A**), nor did it change cell morphology (**B**). 24 h incubation with 10 mM 3-AT significantly reduced ATP content and hence viability of GCs (**C**). All values are shown as mean of three repetitions. Statistics: paired *t*-test with independent measurements (two-tailed; ** p<0.01).

**
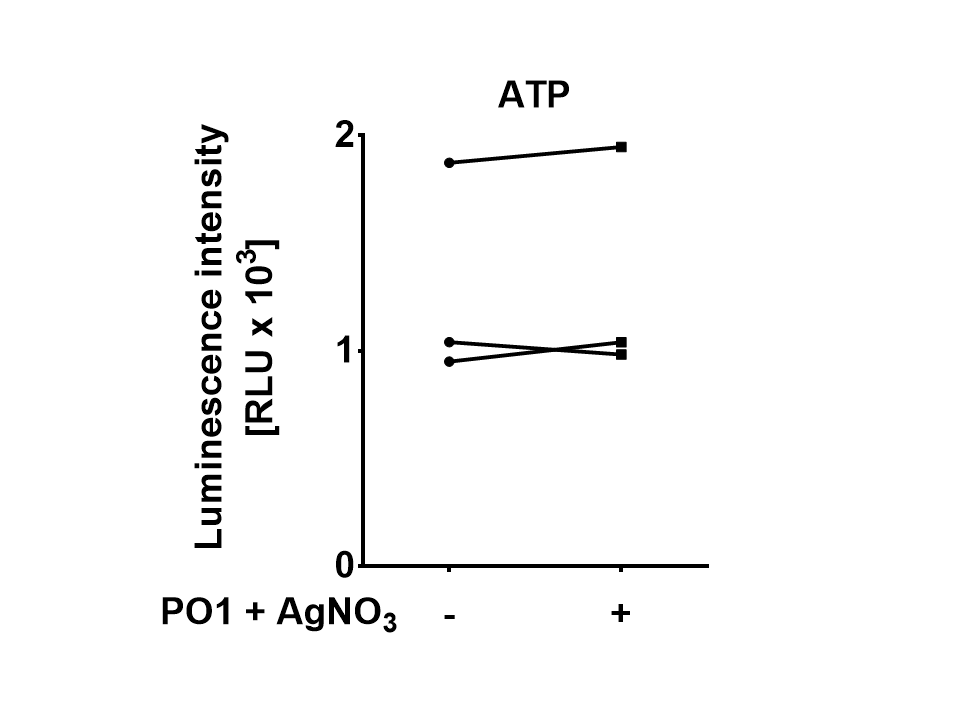
**

**Supplementary Figure S3. AgNO_3_ does not affect viability of GCs.** Treatment (2 h) with AgNO_3_ (500 nM) and PO1 (1 µM) did not affect cell viability of GCs on day 2**.** Shown are ATP values as mean from three measurements. Statistics: paired *t*-test with independent measurements (two-tailed).


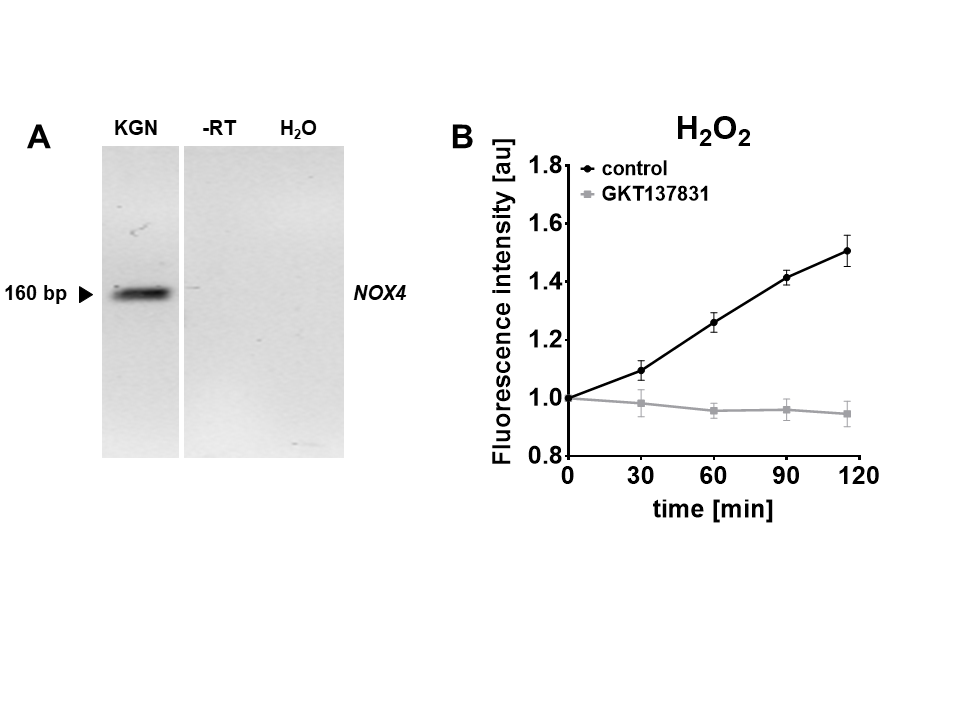


**Supplementary Figure S4. KGN express NOX4 and produce NOX4-derived H_2_O_2_.** (**A**) KGN express *NOX4* confirmed by RT-PCR. Negative controls consist of non-reverse transcribed RNA as template (-RT) and a non-template reaction (H_2_O). (**B**) KGN treated with GKT137831 (20 µM) for 24 h showed a reduction of H_2_O_2_ generation by 45 % compared to control cells (measured by Amplex Red method). Values of one single measurement are shown as mean of six technical repetitions.


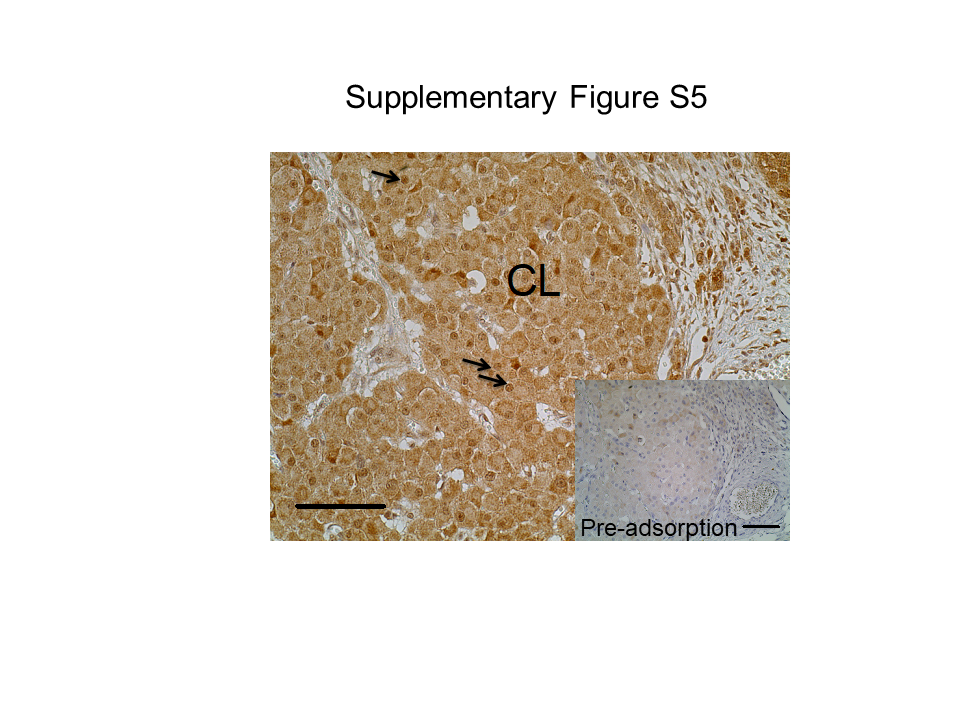


**Supplementary Figure S5. NOX4 presence in CL cells.** (**A**) Cells of corpus luteum of a human ovarian sections are NOX4-positive using immunohistochemistry (anti-NOX4 antibody from Novus Biologicals). Staining is seen intracellularly and in the nucleus (arrows). (**B**) The preadsorption control using the specific peptide is devoid of staining.

**
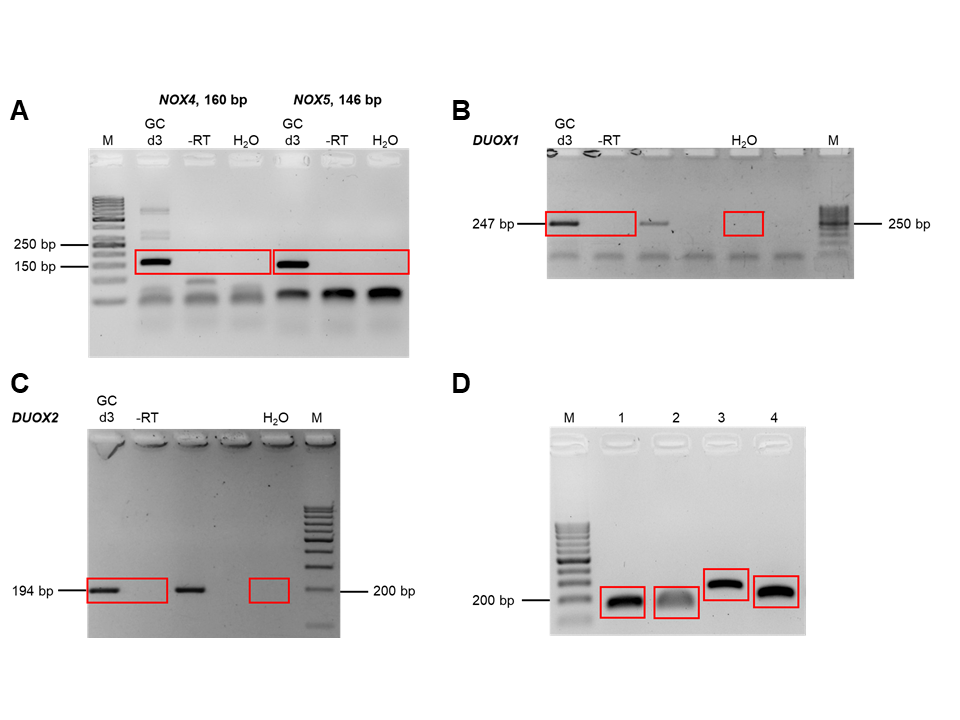
**

**Supplementary Figure S6. Original gel pictures of NOX family expression.** Original pictures of agarose gels for (**A**) *NOX4* and *NOX5*, (**B**) *DUOX1*, (**C**) *DUOX2* on culture day 3, and (**D**) *NOX* and *DUOX* on culture day 0, that were cropped for **Fig. 1A**. (**D**) 1: *NOX4*, 2: *NOX5*, 3: *DUOX1*, 4: *DUOX2*. Cropped parts are marked by red rectangles. M: Thermo Scientific GeneRuler DNA Ladder, -RT: non-reverse transcription control, H_2_O: non-template control.

**
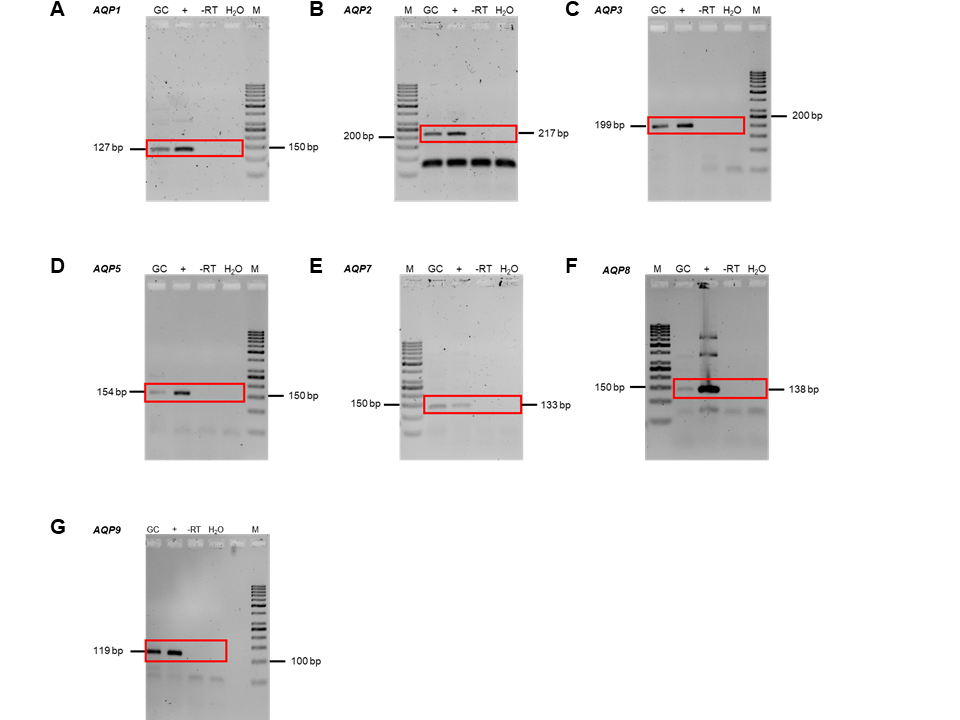
**

**Supplementary Figure S7. Original gel pictures of AQP expression.** Raw data of agarose gels show expression of *AQP1, 2, 3, 5, 7, 8* and *9* (**A-G**), that were cropped for **Fig. 5**. +: positive control (brain/kidney cDNA).

Cropped parts are marked by red rectangles. M: Thermo Scientific GeneRuler DNA Ladder, -RT: non-reverse transcription control, H_2_O: non-template control.


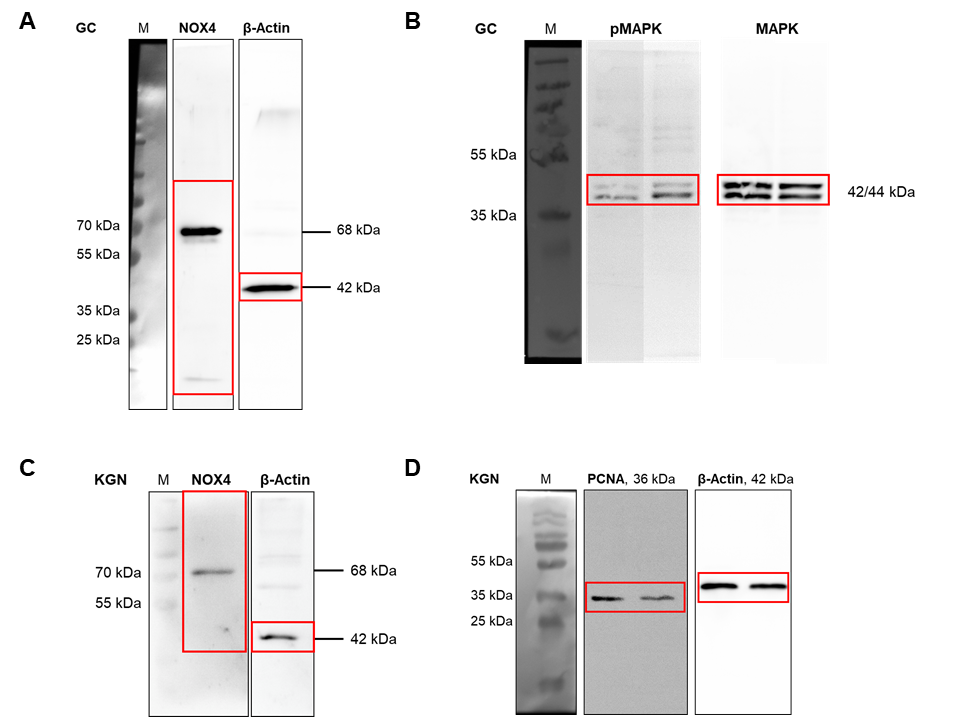


**Supplementary Figure S8. Original pictures from Western blot membranes.** (**A**) NOX4, (**B**) MAPK in GCs, (**C**) NOX4 and (**D**) PCNA in KGN cells, that were cropped for **Fig. 1B**, **6**, **7C** and **Suppl. Fig. 4A**. Cropped parts are marked by red rectangles. M: Thermo Scientific PageRuler Plus Prestained Protein Ladder. Ladder sizes are denoted in [kDa].
